# Longitudinal Associations of Stressful Life Events with Non-suicidal Self-Injury among Chinese Adolescents: The Mediating Effect of Depressive Symptoms

**DOI:** 10.1155/2023/1377714

**Published:** 2023-07-17

**Authors:** Li-Peng Wan, Xiao-Fan Yang, Zhen-Zhen Liu, Bao-Peng Liu, Ying-Ying Zhang, Cai-Rui Liu, Xianchen Liu, Cun-Xian Jia, Xin-Ting Wang

**Affiliations:** ^1^Department of Epidemiology, School of Public Health, Cheeloo College of Medicine, Shandong University and Shandong University Center for Suicide Prevention Research, Jinan, Shandong, China; ^2^School of Psychology, Northeast Normal University, Changchun, Jilin, China; ^3^Center for Public Health Initiatives, University of Pennsylvania, Philadelphia, PA, USA

## Abstract

**Objective:**

Little is known about the mechanism between stressful life events and non-suicidal self-injury (NSSI) in Chinese adolescents. This study was to investigate the mediating effect of depressive symptoms on the association between stressful life events and NSSI among Chinese adolescents.

**Methods:**

This study included a total of 7072 adolescents who participated in the one-year follow-up of Shandong Adolescent Behavior and Health Cohort. A self-administrated questionnaire was used to evaluate stressful life events, depressive symptoms, NSSI, and other variables in November-December 2015. One year later, a follow-up investigation was performed to evaluate participants' depressive symptoms and NSSI. Logistic regression and mediation analysis were used to examine the relationship between stressful life events, depressive symptoms, and NSSI.

**Results:**

Of the sample, half were females and mean age was 14.58 ± 1.46. At baseline and one-year follow-up, the rate of NSSI was 19.4% and 8.8%, respectively. Multivariate logistic regression analysis showed that stressful life events and depressive symptoms were positively associated with NSSI. Mediation analysis showed that depressive symptoms accounted for 17.70% of the relation between high stressful life events and NSSI at one-year follow-up after controlling for covariates. *Limitation.* All variables were collected based on self-report.

**Conclusions:**

The relationship between stressful life events and NSSI appears to be partially mediated by depressive symptoms. It is necessary to evaluate and intervene against depressive symptoms related to life stress for the prevention of NSSI.

## 1. Introduction

Non-suicidal self-injury (NSSI) is the intentional, direct, and self-destruction of bodily tissues with the absence of suicidal intent, where the purpose of the act is not socially acceptable [1, 2]. Cutting, scratching or burning body surfaces, striking objects, and others, causing direct damage to the skin or bones, are common methods of NSSI [3]. NSSI has become a widespread health concern in adolescents across the world. In a meta-analysis published in 2019, the twelve-month prevalence of NSSI was 19.5% globally [4]. A review of NSSI in clinical samples showed that the prevalence of NSSI in the past year ranged from 11.0% to 29.0% among Chinese adolescents [5], and the aggregated estimate of lifetime NSSI prevalence in adolescents was 14.5% [6]. Previous studies revealed that NSSI is strongly associated with future suicidal behavior [7, 8]. For example, in the 13-year follow-up study, the author found that the risk of suicide among individuals who had deliberate self-harm was 37-131 times higher than that of the general population within the first year and 1.7%, 2.4%, and 3.0% after 5, 10, and 15 years of self-injury, respectively [9]. Hence, it is important to study NSSI and its preventable and modifiable factors among adolescents.

Stressful life events are an important component of stressors and have a significant impact on an individual's physical and mental development, and its cumulation might lead to the occurrence of abnormal behavior. Stressful life events are prevalent among adolescents [10], including school-related stressful events (e.g., tension with teachers), family-related stressful events (e.g., serious illness or accidental injury to father or mother), and peer-related stressful events (e.g., tension with friends/classmates) [11]. Children and adolescents are in a rapid and violent stage of physical and mental development, and they are more severely affected by stressful life events compared to adults, so they might do some several harmful behaviors such as NSSI [11].

Previous studies showed that stressful life events were positively associated with NSSI [12–15]. In a study of adolescents with depression, Shao et al. found that higher scores of interpersonal relationships were independently related to NSSI [15]. Another study conducted in a public commuter college found that exposure to stressful life events was positively associated with participating in NSSI thoughts and behaviors [13].

Stressful life events are also risk factors for depression [16]. Based on the stress sensitization model, exposure to stressful life changes during development might enhance adolescent vulnerability to subsequent stressors, further forming depression [17]. The effect of life events needs to be accumulated over the threshold to make people distressed in stress sensitization. Numerous empirical studies also have confirmed that stressful life events are associated with depression [18–20]. In a longitudinal study of adolescents aged 11 to 15 years, Young and Dietrich found that stressful life events were also shown to be predictors of depressive symptoms [21].

Some studies revealed that depressive symptoms are associated with NSSI. For example, Rodav et al. found that depressive symptoms were significantly related to NSSI in 275 Israel adolescents [22]. This was also supported in a systematic review on NSSI and deliberate self-harm, where depressive symptoms were reported to be predictors of NSSI and deliberate self-harm [23]. Some longitudinal studies have also shown that depressive symptoms can predict the occurrence of future NSSI [24, 25]. Moran et al. found that adolescent depressive symptoms were significantly associated with incident NSSI in young adulthood [25].

To the best of our knowledge, most of the research on different environmental risk factors for self-harm has focused on adverse childhood experiences or more severe stressful life events, and the findings on the mediating role of depression between certain types of stressful life events (e.g., childhood abuse and sexual abuse) and NSSI are inconsistent [26, 27]. In a study of Iceland adolescents, Asgeirsdottir et al. found that depressed mood mediated the relationship between sexual abuse and self-injurious behavior as well as the relationship between family conflict/violence and self-injurious behavior [28]. In another study of the relationship between victimization and self-injurious behavior, the authors revealed that depression significantly mediated the relationship between relational victimization and verbal victimization on self-injurious behavior, but it did not mediate the association between physical victimization and self-injurious behavior [26].

**Figure 1:**
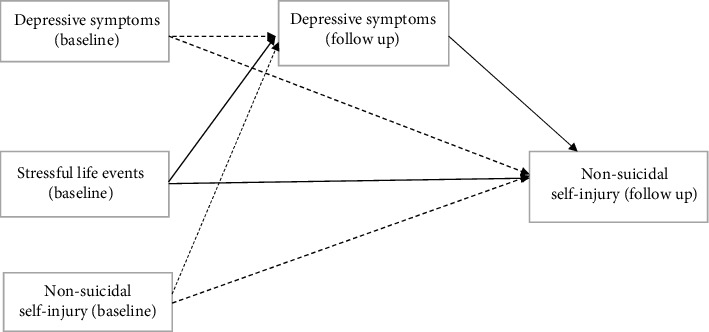
The hypothetical mediating model of depressive symptoms on the association between stressful life events and NSSI.

There are few longitudinal studies examining the relationship between daily stressful life events, depressive symptoms, and NSSI in Chinese adolescents [29, 30]. Using an analysis of 279 Chinese immigrant children, Gao et al. found a temporal association between stressful life events and NSSI, and they found that depressive symptoms mediated the association [29]. Another cross-sectional study conducted among 643 Chinese high school students found that depression mediated the association between stressful life events and NSSI [30]. To our knowledge, no large prospective study has examined the association between a wider variety of stressful life events and NSSI in general Chinese student populations. This study is aimed at exploring the association between the three variables in Chinese adolescents, using longitudinal data from the Shandong Adolescent Behavior and Health Cohort (SABHC). Specifically, this study was to (1) examine the association between daily stressful life events, depressive symptoms, and NSSI and (2) examine whether depressive symptoms could mediate the relationship between daily stressful life events and NSSI. The hypothetical mediation model is shown in Figure 1.

## 2. Methods

### 2.1. Participants

Data for this study were obtained from the Shandong Adolescent Behavior and Health Cohort (SABHC), a three-wave longitudinal study designed to investigate the behavior and health of adolescent students in Shandong Province, China. This cohort was surveyed in 2015 and followed up 1 year later (2016) and 2 years later (2017). Sampling details and data collected can be found in other literature [31–34]. In brief, five middle schools and three high schools in three rural areas (Yanggu, Zoucheng, and Lijin) were sampled to participate in the survey during the baseline period, given the representativeness of the adolescent student population, convenience, and funding to conduct the study. The baseline survey was conducted among 11,831 adolescents from November to December 2015. Since students in grades 9 and 11 did not participate in the follow-up study, only 7,072 children participated in the one-year later follow-up survey. Wave 1 and wave 2 data were used for the current analysis. The flowchart is shown in Supplementary Figure 1.

Adolescent Health Questionnaire (AHQ) was used to collect adolescent students' sociodemographic information, mental health problems, stressful life events, and NSSI. This study gained permission from the principals of the target schools and informed consent from the participants prior to the investigation. It was approved by the Research Ethics Committee of the School of Public Health, Shandong University.

### 2.2. Measures

#### 2.2.1. Demographic Variables

Demographic variables included age, gender, ever drinking (yes or no), ever smoking (yes or no), family economic status (good, fair, or poor), and father's/mother's education level (middle school and below, high school, or college and above).

#### 2.2.2. Non-suicidal Self-Injury (NSSI)

NSSI was defined as a “yes” answer to the question, “In the past 12 months, have you intentionally hurt yourself, but not to kill yourself?”. This question has been applied in many previous studies assessing NSSI in adolescents [31, 33].

#### 2.2.3. Stressful Life Events

Stressful life events in the past year were measured by a modified version of Chinese Adolescent Self-Rating Life Events Checklist (ASLEC) [35, 36]. The modified ASLEC is made up of 50 stressful life events from multiple domains of social stress. Example items include “You lost your love,” “A good friend died,” and “There are frequent conflicts or arguments between parents.” Respondents answered “yes” or “no” to every question regarding whether a specific event happened to him/her in the past 12 months. The total number of stressful life events is the sum of all events experienced in the past 12 months. In the current sample, Cronbach's *α* of the modified version of ASLEC was 0.95 in this sample at baseline. The Kaiser-Meyer-Olkin (KMO) test of this scale was 0.96 (*P* < 0.001). Exploratory factor analysis showed that the cumulative contribution rate of this scale was 53.57% and item loadings of each item were all larger than 0.40, which showed relatively good construct validity. We divided the numbers of life events into three levels: low, *P*_0_ − *P*_50_; moderate, *P*_50_ − *P*_75_; and high, >*P*_75_.

#### 2.2.4. Depressive Symptoms

Depressive symptoms were measured by the Center for Epidemiologic Studies Depression Scale (CES-D) [37]. CES-D, invented by Radloff et al., is a twenty-item self-reported scale used to evaluate a person's depressive symptoms. The answer to each item is based on a four-Likert scale (0 = less than 1 day/week, 1 = one to two days per week, 2 = three to four days/per week, and 3 = five to seven days/week). The total score is calculated by adding up the scores of the 20 items, which may range from 0 to 60. This scale has good internal consistency in adolescents [38, 39]. Cronbach's alpha of this scale was 0.83 at baseline and 0.85 at one-year follow-up with the current sample, respectively. The KMO test of this scale was 0.924 (*P* < 0.001). Exploratory factor analysis showed that the cumulative contribution rate of this scale was 47.82% and item loadings of each item were all larger than 0.40, which showed relatively good construct validity. As reported in previous studies [40, 41], the 90^th^ percentile of CES-D scores for the entire cohort at baseline was used as the cut-off for clinically relevant depressive symptoms (yes = 1, no = 0). Depressive symptoms were surveyed at baseline and one-year follow-up, using the same measurement method.

### 2.3. Statistical Analyses

Continuous variables were described as mean ± SD(SD: standard deviation), and categorical variables were expressed as frequencies (percentages). Chi-square tests and Student's *t*-tests were used to examine differences between individuals with and without NSSI on sociodemographic variables as well as psychological variables. Univariate and multivariate logistic regression were used to compare the relationship between stressful life events, depressive symptoms, and NSSI, expressed as odds ratios (ORs) and their confidence intervals (CIs). In addition, the Karlson-Holm-Breen (KHB) method, which is suitable for mediation analysis of nonlinear models [42], was used to assess the direct effects of stressful life events on subsequent NSSI at one-year follow-up and the mediation role of depressive symptoms in stressful life events-NSSI link. As shown in Figure 1, we performed both unadjusted and adjusted mediation analyses. The unadjusted model was used to examine the relationship between stressful life events and NSSI with depressive symptoms. The adjusted model was then conducted by controlling for the impacts of adolescent and family covariates in Table 1. The KHB method was implemented by the KHB command written by the user in Stata 16.0 [42]. All other analyses were done with SPSS 24.0. Statistical significance was considered when *P* < 0.05.

## 3. Results

### 3.1. Sample Characteristics

Of the 8,629 students in grades 7-8 and 10 at baseline, 7,072 participated in the one-year follow-up survey. At baseline, the mean age of participants was 14.58 (SD: 1.46), and the proportion of females was 50%. The rate of NSSI in the last year was 19.4% at baseline. The rates of low, moderate, and high life stressful events were 35.8%, 26.0%, and 38.2%, respectively, among those with NSSI, compared with 56.3%, 24.1%, and 19.6% among those without NSSI, with differences being statistically significant (*χ*^2^ = 255.38, *P* < 0.001). There was a statistically significant difference in the rate of depressive symptoms among adolescents with NSSI of 21.5% compared to 6.4% among those without NSSI (*χ*^2^ = 294.72, *P* < 0.001).

Table 1 shows the distribution of adolescent and family variables for individuals committing NSSI and those not committing NSSI at baseline. Individuals who committed NSSI were more likely to be older (*t* = −4.46, *P* < 0.001), female (*χ*^2^ = 9.77, *P* = 0.002), ever smoking (*χ*^2^ = 146.64, *P* < 0.001), and ever drinking (*χ*^2^ = 226.31, *P* < 0.001). There were statistically significant differences between people with NSSI and without NSSI in family economic status (*χ*^2^ = 15.05, *P* < 0.001), but not in father's and mother's education level (all *P* values > 0.05). The correlation between study variables is shown in Supplementary Table 2.

### 3.2. Association between Stressful Life Events, Depressive Symptoms, and NSSI in a One-Year Follow-Up

Table 2 presents the association between stressful life events, depressive symptoms, and NSSI at a one-year follow-up. At a one-year follow-up, the rate of NSSI was 8.8%. Univariate analysis showed that adolescents with moderate (OR = 1.33, 95% CI = 1.08 − 1.64) and high stressful life events (OR = 2.18, 95% CI = 1.80 − 2.63) were more likely to commit NSSI compared with individuals with low stressful life events; depressive symptoms were positively associated with NSSI (OR = 5.15, 95% CI = 4.10 − 6.48). After controlling for adolescents and family covariates, only high stressful life events were associated with with NSSI (OR = 1.28, 95% CI = 1.02 − 1.59) and the OR of depressive symptoms was reduced (OR = 3.55, 95% CI = 2.73 − 4.61) and statistically significant (*P* < 0.05).

### 3.3. Mediating Role of Depressive Symptoms

Table 3 presents path coefficients and 95% confidence interval for NSSI associated with stressful life events, with depressive symptoms as the mediating factor. The log-odd of NSSI was significantly higher among adolescents who had high stressful life events compared to those who had low stressful life events. When predicting NSSI from stressful life events without controlling covariates, the indirect and direct coefficients for moderate and high stressful life events were significant (*P* values < 0.05), indicating a partial mediation relationship for NSSI. After controlling for covariates, the magnitude of indirect effects from high stressful life events to NSSI via depressive symptoms changed from 0.120 (95% CI = 0.086 − 0.155) to 0.052 (95% CI = 0.025 − 0.079). The magnitude of direct coefficient of high stressful life events to NSSI changed from 0.610 (95% CI = 0.412 − 0.807) to 0.242 (95% CI = 0.022 − 0.463). The mediation proportion changed from 16.48% to 17.70% for high stressful life events. After further controlling follow-up stressful life events, depressive symptoms still play a mediating role (see Supplementary Table 3).

## 4. Discussions

To our knowledge, this is one of the largest studies examining the relationship between stressful life events, depressive symptoms, and NSSI in Chinese adolescents. In this study, we found the following: (1) both stressful life events and depressive symptoms were positively associated with NSSI; (2) depressive symptoms partially mediated the relationship between stressful life events and NSSI.

In our study, we found a prevalence of 19.4% and 8.8% of NSSI at baseline and one year later, respectively. At baseline, participants were 7th graders (that is, the first year of middle school) or 10th graders (that is, high school). The new school environment as well as the added academic pressure can be stressful and result in emotional dysregulation for 7th graders or 10th graders [43]. This, in turn, increased the risk of NSSI. At the time of follow-up, students may have adjusted to the school environment and have a lower risk of NSSI.

Stressful life events, as events that tend to trigger emotional dysregulation in individuals, often involve changes in physical health status, personal lifestyle, or behavior and may also involve successes or failures that are significant to the individual. In our sample, we found that stressful life events were positively related to NSSI. This association has been demonstrated by many cross-sectional and longitudinal studies in special and general populations [15, 44–46]. In a cross-sectional study of adolescents with depression, Shao et al. found that interpersonal relationships were positively associated with NSSI [15]. In another study of male justice-invoked adolescents, Drubina et al. found that negative romantic relationship was a risk factor for NSSI [45]. The relationship might be explained by the cumulative effect of negative life events on an individual's health, meaning that over time, people who experience multiple negative life events are likely to experience more adverse health consequences [47].

It was also found that depressive symptoms were a significant factor for NSSI, consistent with previous studies [22, 24, 25, 48]. In this association, emotion regulation is most often seen as a motivating factor or function of self-jury [48], which might lead to taking NSSI to get rid of these emotions, when individuals have depressive symptoms [49].

Consistent with Gao et al.'s study of migrant adolescents [29], our mediation analysis found that the relationship between stressful life events and NSSI was mediated by depressive symptoms. This finding was supported by several studies that examined the effect of depressive symptoms as a mediator on negative life events and self-injuries [27, 28]. Exposure to stressful life changes during development, according to the stress sensitization model, enhances vulnerability to subsequent stressors, further leading to depressive symptoms [17]. And NSSI may be used to get rid of or escape from depressive symptoms [49]. Besides, depressive symptoms playing a mediating role are also in accordance with the general strain theory of deviance, of which the main premise is that strained relationships and events force individuals to commit deviant behaviors through psychological stress or negative affect [50]. Agnew considered that individuals who develop depression to cope with stress are more likely to commit self-directed behaviors, like suicidal behavior and NSSI [51]. Based on the experiential avoidance model of NSSI [52], an individual undergoes a negative event that evokes strong, distressing emotions. As a response, an individual has the impulse to avoid the distressful emotional state and may adopt NSSI as a means of reducing or eliminating the emotional arousal. Many studies have shown that exposure to high numbers of stress events can lead to the incidence of depression [53, 54]. Thus, for adolescents with their own depressive symptoms, high levels of exposure to life events can increase the level of depression even more, which in turn increases risk of NSSI.

This research may have important implications for NSSI intervention and prevention. Mental health practitioners should screen adolescents who have experienced high levels of stressful life events, which can be done by using a number of stressful life event scales. And those exposed to some stressful life events should be viewed as a group that is particularly vulnerable and needs more attention. The government and clinicians should realize that stressful life events contribute to increasing NSSI, partly by depressive symptoms. Meanwhile, providing people with alternative, healthy emotion management strategies may be an effective way to prevent them from engaging in NSSI to respond to depressed mood. Schools and social workers should offer a number of intervention programs such as sports training and positive thought-based interventions. Teachers and parents should be alert to potentially stressful events and depressive symptoms in the lives of adolescents' regular life and provide diversion and assistance for problems they countered. Furthermore, schools should consider mental health programs to help students with managing stressful life events and negative emotion to reduce the risk of depression.

## 5. Limitations

The study has several limitations. First, the study used a self-reported method to collect data, which may result in information bias. Second, the study investigated middle and high school students in Shandong Province, so whether it can be generalized to other regions and other populations remains unknown. Third, other potential confounding factors such as anxiety and externalizing problems could not be included. Finally, the follow-up was only once and only one year.

## 6. Conclusion

Using a longitudinal study of a large sample of Chinese adolescents, we demonstrated that the relationship between stressful life events and NSSI was partially mediated by depressive symptoms. These findings require clinicians, teachers, and parents to pay special attention to adolescents exposed to stressful life events, and depressive symptoms need appropriate intervention to reduce the risk of NSSI.

## Figures and Tables

**Table 1:** Sample characteristics by last-year NSSI at baseline.

Variables	Total (*n* = 7072)	NSSI (*n* = 1374)	Non-NSSI (*n* = 5698)	*χ* ^2^/*t*	*P*
Stressful life events, *n* (%)				255.38	<0.001
Low	3699 (52.3)	492 (35.8)	3207 (56.3)		
Moderate	1731 (24.5)	357 (26.0)	1374 (24.1)		
High	1642 (23.2)	525 (38.2)	1117 (19.6)		
Depressive symptoms, *n* (%)				294.72	<0.001
Yes	662 (9.4)	295 (21.5)	367 (6.4)		
No	6410 (90.6)	1079 (78.5)	5331 (93.6)		
Age, mean (SD)	14.58 (1.46)	14.74 (1.39)	14.54 (1.47)	-4.46	<0.001
Gender, *n* (%)				9.77	0.002
Male	3536 (50.0)	635 (46.2)	2901 (50.9)		
Female	3536 (50.0)	739 (53.8)	2797 (49.1)		
Ever smoking, *n* (%)				146.64	<0.001
Yes	1339 (18.9)	418 (30.4)	921 (16.2)		
No	5733 (81.1)	956 (69.6)	4777 (83.8)		
Ever drinking, *n* (%)				226.31	<0.001
Yes	2417 (34.2)	707 (51.5)	1710 (30.0)		
No	4655 (65.8)	667 (48.5)	3988 (70.0)		
Family economic status, *n* (%)				15.05	0.001
Good	1444 (20.4)	264 (19.2)	1180 (20.7)		
Fair	4786 (67.7)	905 (65.9)	3881 (68.1)		
Poor	842 (11.9)	205 (14.9)	637 (11.2)		
Father's education level, *n* (%)				0.82	0.664
Middle school and below	4827 (68.3)	924 (67.2)	3903 (68.5)		
High school	1304 (18.4)	260 (18.9)	1044 (18.3)		
College and above	941 (13.3)	190 (13.8)	751 (13.2)		
Mother's education level, *n* (%)				1.80	0.407
Middle school and below	5399 (76.3)	1039 (75.6)	4360 (76.5)		
High school	918 (13.0)	193 (14.0)	725 (12.7)		
College and above	755 (10.7)	142 (10.3)	613 (10.8)		

**Table 2:** Association between stressful life events, depressive symptoms, and NSSI in a one-year follow-up.

Variables	Model 1			Model 2		
	OR	95% CI	*P*	OR	95% CI	*P*
Stressful life events at baseline						
Low	1			1		
Moderate	1.33	1.08-1.64	0.006	1.07	0.85-1.34	0.558
High	2.18	1.80-2.63	<0.001	1.28	1.02-1.59	0.031
Depressive symptoms at follow-up						
No	1			1		
Yes	5.15	4.10-6.48	<0.001	3.55	2.73-4.61	<0.001

Model 1: univariate logistic analysis. Model 2: multivariate logistic analysis, controlling for covariates in Table 1 except stressful life events+NSSI at baseline. CI: confidence interval; NSSI: non-suicidal self-injury.

**Table 3:** Mediating effect of depressive symptoms on the relationship between stressful life events and NSSI.

	Unadjusted model			Adjusted model		
	Effect	95% CI	% mediated	Effect	95% CI	% mediated
Stressful life events (ref = low)						
Moderate			13.37			8.46
Total effect	0.265⁣^∗^	0.052-0.479		0.073	-0.153-0.299	
Direct effect	0.230⁣^∗^	0.016-0.443		0.067	-0.160-0.293	
Indirect effect	0.035⁣^∗^	0.006-0.065		0.006	-0.018-0.031	
High			16.48			17.70
Total effect	0.730⁣^∗∗∗^	0.535-0.925		0.294⁣^∗^	0.074-0.514	
Direct effect	0.610⁣^∗∗∗^	0.412-0.807		0.242⁣^∗^	0.022-0.463	
Indirect effect	0.120⁣^∗∗∗^	0.086-0.155		0.052⁣^∗∗∗^	0.025-0.079	

Unadjusted model: predicting NSSI from stressful life events with depressive symptoms at one-year follow-up being the mediator. Adjusted model: unadjusted model+controlling for covariates in Table 1 except for stressful life events+NSSI at baseline. ⁣^∗^*P* < 0.05,⁣^∗∗^*P* < 0.01, and⁣^∗∗∗^*P* < 0.001. CI: confidence interval; NSSI: non-suicidal self-injury.

## Data Availability

The data that support the findings of this study are available from the corresponding authors upon reasonable request.

## Supplementary Materials

Supplementary Materials Supplementary Figure 1: flowchart of the study. Supplementary Table 1: the differences between populations with different status NSSI. Supplementary Table 2: the correlation between study variables. Supplementary Table 3: mediating effect of depressive symptoms on the relationship between stressful life events and NSSI (additional adjustments to follow-up stressful life events).

## References

[1] Liu R. T., Scopelliti K. M., Pittman S. K., Zamora A. S. (2018). Childhood maltreatment and non-suicidal self-injury: a systematic review and meta-analysis. *Lancet Psychiatry*.

[2] Nock M. K., Favazza A. R., Nock M. K. (2009). Non-suicidal self-injury: Definition and classification. *Understanding Non-suicidal Self-Injury: Origins, Assessment, and Treatment*.

[3] Plener P. L., Kaess M., Schmahl C., Pollak S., Fegert J. M., Brown R. C. (2018). Non-suicidal self-injury in adolescents. *Deutsches Arzteblatt International*.

[4] Lim K. S., Wong C. H., McIntyre R. S. (2019). Global lifetime and 12-month prevalence of suicidal behavior, deliberate self-harm and non-suicidal self-injury in children and adolescents between 1989 and 2018: a meta-analysis. *International Journal of Environmental Research and Public Health*.

[5] Swannell S. V., Martin G. E., Page A., Hasking P., St John N. J. (2014). Prevalence of non-suicidal self-injury in nonclinical samples: systematic review, meta-analysis and meta-regression. *Suicide and Life-threatening Behavior*.

[6] Lang J. J., Yao Y. S. (2018). Prevalence of non-suicidal self-injury in Chinese middle school and high school students a meta-analysis. *Medicine*.

[7] Kiekens G., Hasking P., Boyes M. (2018). The associations between non-suicidal self-injury and first onset suicidal thoughts and behaviors. *Journal of Affective Disorders*.

[8] Pérez S., Ros M. C., Layron Folgado J. E., Marco J. H. (2019). Non-suicidal self-injury differentiates suicide ideators and attempters and predicts future suicide attempts in patients with eating disorders. *Suicide & Life-Threatening Behavior*.

[9] Hawton K., Zahl D., Weatherall R. (2003). Suicide following deliberate self-harm: long-term follow-up of patients who presented to a general hospital. *The British Journal of Psychiatry*.

[10] Barnes V. A., Bauza L. B., Treiber F. A. (2003). Impact of stress reduction on negative school behavior in adolescents. *Health and Quality of Life Outcomes*.

[11] Zhao F., Zhang Z. H., Bi L. (2017). The association between life events and internet addiction among Chinese vocational school students: the mediating role of depression. *Computers in Human Behavior*.

[12] Crowell S. E., Baucom B. R., McCauley E. (2013). Mechanisms of contextual risk for adolescent self-injury: invalidation and conflict escalation in mother-child interactions. *Journal of Clinical Child and Adolescent Psychology*.

[13] Macrynikola N., Miranda R., Soffer A. (2018). Social connectedness, stressful life events, and self-injurious thoughts and behaviors among young adults. *Comprehensive Psychiatry*.

[14] Madge N., Hawton K., McMahon E. M. (2011). Psychological characteristics, stressful life events and deliberate self-harm: findings from the child & adolescent self-harm in Europe (CASE) study. *European Child & Adolescent Psychiatry*.

[15] Shao C., Wang X., Ma Q., Zhao Y., Yun X. (2021). Analysis of risk factors of non-suicidal self-harm behavior in adolescents with depression. *Annals of Palliative Medicine*.

[16] Cohen S., Murphy M. L. M., Prather A. A. (2019). Ten surprising facts about stressful life events and disease risk. *Annual Review of Psychology*.

[17] Hammen C., Henry R., Daley S. E. (2000). Depression and sensitization to stressors among young women as a function of childhood adversity. *Journal of Consulting and Clinical Psychology*.

[18] Infurna M. R., Reichl C., Parzer P., Schimmenti A., Bifulco A., Kaess M. (2016). Associations between depression and specific childhood experiences of abuse and neglect: a meta-analysis. *Journal of Affective Disorders*.

[19] Kendler K. S., Karkowski L. M., Prescott C. A. (1999). Causal relationship between stressful life events and the onset of major depression. *American Journal of Psychiatry*.

[20] Tessner K. D., Mittal V., Walker E. F. (2011). Longitudinal study of stressful life events and daily stressors among adolescents at high risk for psychotic disorders. *Schizophrenia Bulletin*.

[21] Young C. C., Dietrich M. S. (2015). Stressful life events, worry, and rumination predict depressive and anxiety symptoms in young adolescents. *Journal of Child and Adolescent Psychiatric Nursing*.

[22] Rodav O., Levy S., Hamdan S. (2014). Clinical characteristics and functions of non-suicide self-injury in youth. *European Psychiatry*.

[23] Plener P. L., Schumacher T. S., Munz L. M., Groschwitz R. C. (2015). The longitudinal course of non-suicidal self-injury and deliberate self-harm: a systematic review of the literature. *Borderline Personality Disorder and Emotion Dysregulation*.

[24] Hankin B. L., Abela J. R. Z. (2011). Non-suicidal self-injury in adolescence: prospective rates and risk factors in a 2 ½ year longitudinal study. *Psychiatry Research*.

[25] Moran P., Coffey C., Romaniuk H. (2012). The natural history of self-harm from adolescence to young adulthood: a population-based cohort study. *Lancet*.

[26] Brunstein Klomek A., Snir A., Apter A. (2016). Association between victimization by bullying and direct self injurious behavior among adolescence in Europe: a ten-country study. *European Child & Adolescent Psychiatry*.

[27] Shenk C. E., Noll J. G., Cassarly J. A. (2010). A multiple mediational test of the relationship between childhood maltreatment and non-suicidal self-injury. *Journal of Youth and Adolescence*.

[28] Asgeirsdottir B. B., Sigfusdottir I. D., Gudjonsson G. H., Sigurdsson J. F. (2011). Associations between sexual abuse and family conflict/violence, self-injurious behavior, and substance use: the mediating role of depressed mood and anger. *Child Abuse and Neglect*.

[29] Gao Y. M., Wang H., Liu X., Xiong Y., Wei M. (2020). Associations between stressful life events, non-suicidal self-injury, and depressive symptoms among Chinese rural-to-urban children: a three-wave longitudinal study. *Stress and Health*.

[30] Wei C., Li Z., Ma T., Jiang X., Yu C., Xu Q. (2022). Stressful life events and non-suicidal self-injury among Chinese adolescents: a moderated mediation model of depression and resilience. *Frontiers in Public Health*.

[31] Liu X. C., Chen H., Bo Q. G., Fan F., Jia C. X. (2017). Poor sleep quality and nightmares are associated with non-suicidal self-injury in adolescents. *European Child & Adolescent Psychiatry*.

[32] Liu X. C., Chen H., Liu Z. Z., Wang J. Y., Jia C. X. (2019). Prevalence of suicidal behaviour and associated factors in a large sample of Chinese adolescents. *Epidemiology and Psychiatric Sciences*.

[33] Liu Z. Z., Chen H., Bo Q. G. (2018). Psychological and behavioral characteristics of suicide attempts and non- suicidal self-injury in Chinese adolescents. *Journal of Affective Disorders*.

[34] Wan L. P., Yang X. F., Liu B. P. (2022). Depressive symptoms as a mediator between perceived social support and suicidal ideation among Chinese adolescents. *Journal of Affective Disorders*.

[35] Liu X. C., Kurita H., Uchiyama M., Okawa M., Liu L., Ma D. (2000). Life events, locus of control, and behavioral problems among Chinese adolescents. *Journal of Clinical Psychology*.

[36] Liu X. C., Tein J. Y. (2005). Life events, psychopathology, and suicidal behavior in Chinese adolescents. *Journal of Affective Disorders*.

[37] Radloff L. S. (1977). The CES-D scale. *Applied Psychological Measurement*.

[38] Betancourt T., Scorza P., Meyers-Ohki S. (2012). Validating the center for epidemiological studies depression scale for children in Rwanda. *Journal of the American Academy of Child and Adolescent Psychiatry*.

[39] Heo E. H., Choi K. S., Yu J. C., Nam J. A. (2018). Validation of the center for epidemiological studies depression scale among Korean adolescents. *Psychiatry Investigation*.

[40] Hu L., Liu Z. Z., Wang Z. Y., Jia C. X., Liu X. (2022). Associations between pain and depressive symptoms: a longitudinal study of Chinese adolescents. *Journal of Affective Disorders*.

[41] Liu B. P., Wang X. T., Liu Z. Z. (2020). Depressive symptoms are associated with short and long sleep duration: a longitudinal study of Chinese adolescents. *Journal of Affective Disorders*.

[42] Kohler U., Karlson K. B., Holm A. (2011). Comparing coefficients of nested nonlinear probability models. *Stata Journal*.

[43] Benner A. D., Graham S. (2009). The transition to high school as a developmental process among multiethnic urban youth. *Child Development*.

[44] Baetens I., Greene D., van Hove L. (2021). Predictors and consequences of non-suicidal self-injury in relation to life, peer, and school factors. *Journal of Adolescence*.

[45] Drubina B., Kökönyei G., Reinhardt M. (2021). Associations between non-suicidal self-injury and negative romantic relationship life events in male justice-involved adolescents. *BMC Psychiatry*.

[46] Steinhoff A., Bechtiger L., Ribeaud D., Eisner M., Shanahan L. (2020). Stressful life events in different social contexts are associated with self-injury from early adolescence to early adulthood. *Frontiers in Psychiatry*.

[47] Kanner A. D., Coyne J. C., Schaefer C., Lazarus R. S. (1981). Comparison of two modes of stress measurement: daily hassles and uplifts versus major life events. *Journal of Behavioral Medicine*.

[48] Nixon M. K., Cloutier P. F., Aggarwal S. (2002). Affect regulation and addictive aspects of repetitive self-injury in hospitalized adolescents. *Journal of the American Academy of Child and Adolescent Psychiatry*.

[49] Klonsky E. D. (2007). The functions of deliberate self-injury: a review of the evidence. *Clinical Psychology Review*.

[50] Agnew R. (1992). Foundation for a general strain theory of crime and delinquency. *Criminology*.

[51] Agnew R. (2001). Building on the foundation of general strain theory: specifying the types of strain most likely to lead to crime and delinquency. *Journal of Research in Crime and Delinquency*.

[52] Chapman A. L., Gratz K. L., Brown M. Z. (2006). Solving the puzzle of deliberate self-harm: the experiential avoidance model. *Behaviour Research and Therapy*.

[53] McLaughlin K. A., Conron K. J., Koenen K. C., Gilman S. E. (2010). Childhood adversity, adult stressful life events, and risk of past-year psychiatric disorder: a test of the stress sensitization hypothesis in a population-based sample of adults. *Psychological Medicine*.

[54] Suliman S., Mkabile S. G., Fincham D. S., Ahmed R., Stein D. J., Seedat S. (2009). Cumulative effect of multiple trauma on symptoms of posttraumatic stress disorder, anxiety, and depression in adolescents. *Comprehensive Psychiatry*.

