# Supplementary material for: Longitudinal Associations of Stressful Life Events with Non-suicidal Self-Injury among Chinese Adolescents: The Mediating Effect of Depressive Symptoms
Source: Depress Anxiety. 2023 Jul 17;2023:1377714. doi: 10.1155/2023/1377714 (PMC11921865; doi:10.1155/2023/1377714)
Supplement: Supplementary Materials — Supplementary Figure 1: flowchart of the study. Supplementary Table 1: the differences between populations with different status NSSI. Supplementary Table 2: the correlation between study variables. Supplementary Table 3: mediating effect of depressive symptoms on the relationship between stressful life events and NSSI (additional adjustments to follow-up stressful life events). [file 1377714.f1.docx]

Baseline survey, n=11831 (2015)

grade 9 and 11 didn’t participate follow-up study

Follow-up survey, n=7072 (2016)

Sample characteristics

The mediating role of depressive symptoms on the association between stressful life events and NSSI at one-year later

Association between stressful life events, depressive symptoms, and NSSI at one-year later

Supplementary Figure 1 Flowchart of the study

The number of participants who did have experience NSSI was 1374. Among those who engaged in NSSI at baseline, the proportion of those who did NSSI at follow-up was 23.07% (317/1374). Of those who didn’t do NSSI at baseline, 5.34% (304/5698) engaged in NSSI at follow-up. We found that the differences in the distribution of stressful life events at baseline and depressive symptoms at baseline and follow-up between the different status NSSI populations were statistically significant (*P*< 0.001).

Supplementary Table 1 The Differences between populations with different status NSSI.

|  | Baseline non-NSSI + follow-up non-NSSI (n=5394) | Baseline NSSI + follow-up non-NSSI (n=1057) | Baseline non-NSSI + follow-up NSSI (n=304) | Baseline NSSI + follow-up NSSI (n=317) | *χ^2^* | *P* |
| --- | --- | --- | --- | --- | --- | --- |
| Stressful life events (%) |  |  |  |  | 274.35 | <0.001 |
| low | 3057 (56.67) | 394 (37.28) | 150 (49.34) | 98 (30.91) |  |  |
| moderate | 1304 (24.18) | 276 (26.11) | 70 (23.03) | 81 (25.55) |  |  |
| high | 1033 (19.15) | 387 (36.61) | 84 (27.63) | 138 (45.53) |  |  |
| Depressive symptoms at baseline |  |  |  |  | 344.94 | <0.001 |
| yes | 332 (6.15) | 198 (18.73) | 35 (11.51) | 97 (30.60) |  |  |
| no | 5062 (93.85) | 859 (81.27) | 269 (88.49) | 220 (69.40) |  |  |
| Depressive symptoms at follow-up |  |  |  |  | 271.27 | <0.001 |
| yes | 208 (3.86) | 87 (8.23) | 54 (17.76) | 69 (21.77) |  |  |
| no | 5186 (96.14) | 970 (91.77) | 250 (82.24) | 248 (78.23) |  |  |

Supplementary Table 2 The correlation between study variables

|  | 1 | 2 | 3 | 4 | 5 | 6 | 7 | 8 | 9 | 10 | 11 | 12 |
| --- | --- | --- | --- | --- | --- | --- | --- | --- | --- | --- | --- | --- |
| 1.gender ^a^ | 1.000 | 0.042^**^ | 0.244^**^ | 0.219^**^ | 0.029^*^ | - 0.022 | - 0.024^*^ | - 0.028^*^ | 0.038^**^ | 0.031^**^ | 0.037^**^ | 0.044^**^ |
| 2.age at baseline ^b^ |  | 1.000 | - 0.136^**^ | - 0.243^**^ | 0.133^**^ | 0.067^**^ | 0.000 | 0.192^**^ | 0.099^**^ | 0.051^**^ | 0.052^**^ | - 0.014 |
| 3.Ever smoking at baseline |  |  | 1.000 | 0.364^**^ | - 0.050^**^ | 0.044^**^ | 0.066^**^ | - 0.175^**^ | - 0.094^**^ | - 0.055^**^ | - 0.144^**^ | - 0.076^**^ |
| 4.Ever drinking at baseline |  |  |  | 1.000 | - 0.066^**^ | - 0.019 | 0.003 | - 0.213^**^ | - 0.103^**^ | - 0.071^**^ | - 0.179^**^ | - 0.102^**^ |
| 5.Family economic status at baseline |  |  |  |  | 1.000 | - 0.212^**^ | - 0.214^**^ | 0.146^**^ | 0.097^**^ | 0.067^**^ | 0.036^**^ | 0.005 |
| 6.Father education level at baseline |  |  |  |  |  | 1.000 | 0.569^**^ | - 0.060^**^ | - 0.006 | 0.006 | 0.011 | - 0.020 |
| 7.Mother education level at baseline |  |  |  |  |  |  | 1.000 | - 0.089^**^ | - 0.020 | 0.000 | 0.006 | - 0.005 |
| 8.Stressful life events |  |  |  |  |  |  |  | 1.000 | 0.203^**^ | 0.128^**^ | 0.185^**^ | 0.091^**^ |
| 9.Depressive symptoms at baseline |  |  |  |  |  |  |  |  | 1.000 | 0.265^**^ | 0.204^**^ | 0.127^**^ |
| 10.Depressive symptoms at one-year follow-up |  |  |  |  |  |  |  |  |  | 1.000 | 0.113^**^ | 0.183^**^ |
| 11. NSSI at baseline |  |  |  |  |  |  |  |  |  |  | 1.000 | 0.248^**^ |
| 12. NSSI at one-year follow-up |  |  |  |  |  |  |  |  |  |  |  | 1.000 |

^a^ male=0, female=1

^b^ The mean and standard deviation of age at baseline was 14.58 and 1.46.

*P* < 0.05 ^*^, *P* < 0.01^**^, *P* < 0.001^***^

Supplementary Table 3 Mediating effect of depressive symptoms on the relationship between stressful life events and NSSI (Additional adjustments to follow-up stressful life events)

| Stressful life events (ref = low) | *β* | *SE*(*β*) | *Z* | *P* | 95% *CI* | % mediated |
| --- | --- | --- | --- | --- | --- | --- |
| Moderate |  |  |  |  |  | 85.27% |
| Total effect | 0.126 | 0.116 | 1.08 | 0.279 | -0.102-0.354 |  |
| Direct effect | 0.019 | 0.116 | 0.16 | 0.874 | -0.210-0.247 |  |
| Indirect effect | 0.107 | 0.025 | 4.21 | < 0.001 | 0.057-0.157 |  |
| High |  |  |  |  |  | 68.82% |
| Total effect | 0.368 | 0.112 | 3.29 | 0.001 | 0.149-0.588 |  |
| Direct effect | 0.115 | 0.114 | 1.01 | 0.312 | -0.108-0.338 |  |
| Indirect effect | 0.254 | 0.030 | 8.54 | < 0.001 | 0.195-0.312 |  |

Adjusted model: unadjusted model + controlling for covariates in table 1 except for stressful life events+ NSSI at baseline+ stressful life events in Time 2.
